# Optimizing pregnant mare serum gonadotropin dosage for reproductive efficiency and lamb survival in Dorper × Assaf ewes during the non-breeding season

**DOI:** 10.14202/vetworld.2025.3561-3570

**Published:** 2025-11-27

**Authors:** Wael Halawa, Ikram Bensouf, Samia Khnissi, Muayad Salman, Musa Khaleel, Naceur M’Hamdi

**Affiliations:** 1 Laboratory of Animal, Genetic and Feed Resources, National Agronomic Institute of Tunisia, University of Carthage, Carthage 1054, Tunisia; 2 Laboratory of Animal and Forage Production, National Institute of Agronomic Research of Tunisia, University of Carthage, Tunis 1054, Tunisia; 3 National Agricultural Research Center, Nablus, Jenin Street, Palestinian Ministry of Agriculture, Palestine

**Keywords:** Animal welfare, Dorper ×, Assaf ewes, estrus synchronization, lamb survival, pregnant mare serum gonadotropin dosage, reproductive efficiency, sustainable development goals 12, sustainable development goals 2

## Abstract

**Background and Aim:**

Reproductive efficiency during the non-breeding season is a critical factor influencing year-round productivity in sheep farming, particularly in semi-arid environments. Pregnant mare serum gonadotropin (PMSG) is commonly used to induce estrus, yet the optimal dosage that maximizes fertility and lamb growth while minimizing hormonal side effects remains unclear. This study aimed to determine the optimal PMSG dose for enhancing reproductive and growth performance in Dorper × Assaf crossbred ewes under semi-extensive conditions in Palestine.

**Materials and Methods:**

A total of 143 non-lactating Dorper × Assaf ewes (aged 2–3 years) were synchronized with intravaginal progestagen sponges for 14 days, followed by intramuscular administration of 400, 500, or 600 international units (IU) of PMSG. Key reproductive traits, estrus response, conception rate, abortion rate, fecundity, and lamb survival, were recorded, alongside lamb birth and weaning weights. Data were analyzed using a one-way analysis of variance and a general linear model incorporating parity, litter size, and sex effects, with significance set at p < 0.05.

**Results:**

High conception and lambing rates were observed across all groups. The 400 IU PMSG dose resulted in the lowest abortion rate (2.44%) and highest lamb survival (96.9%), while higher doses (500–600 IU) increased abortion incidence without improving fertility outcomes. Birth weight increased with PMSG level (3.42–3.83 kg), whereas weaning weight peaked at 500 IU (22.18 kg). Litter size and lamb sex significantly affected both birth and weaning weights, with singletons and males being heavier.

**Conclusion:**

Administering 400 IU of PMSG provides the optimal balance between reproductive performance, lamb survival, cost-efficiency, and animal welfare. Excessive hormonal stimulation at higher doses offers no additional reproductive benefit and may compromise ewe health. Implementing this moderate, breed-specific hormonal protocol enhances fertility while reducing veterinary intervention and production costs, promoting sustainability and welfare-conscious management. These outcomes directly contribute to sustainable development goals (SDG) 2 – zero hunger and SDG 12 – responsible consumption and production, advancing resilient and ethical small-ruminant farming systems.

## INTRODUCTION

Reproductive efficiency is a critical determinant of productivity in sheep farming, directly influencing both economic profitability and genetic improvement [1]. Effective reproductive management is essential for sustaining lamb production throughout the year, particularly under semi-intensive systems prevalent in arid and semi-arid regions [2]. To overcome the reproductive seasonality of ewes, estrus induction and synchronization during the non-breeding season are commonly achieved using intravaginal progestagen sponges followed by equine chorionic gonadotropin (eCG, formerly pregnant mare serum gonadotropin [PMSG]) administration. This approach facilitates out-of-season breeding, ensuring a continuous lamb supply and optimizing flock productivity in breeds such as Assaf sheep [3].

Estrus induction during seasonal anestrus typically involves the insertion of intravaginal progestagen-impregnated sponges or controlled internal drug release devices for 12–14 days. On removal, eCG is administered to stimulate follicular development, ovulation, and fertile estrus, due to its combined follicle-stimulating hormone- and luteinizing hormone-like activities. This hormone-assisted synchronization protocol is among the most reliable and widely adopted in small ruminant reproduction [4, 5]. Nevertheless, the optimal eCG dose may vary with breed, physiological status, management system, and environmental factors.

Breed-specific variations in hormonal responsiveness further complicate the establishment of universal protocols, underscoring the need for tailored reproductive management strategies [6]. The Dorper × Assaf crossbred ewe, increasingly adopted in Palestine, integrates the high fertility and adaptability of the Dorper with the dual-purpose productivity and resilience of the Assaf breed [7, 8]. Despite its growing importance, limited information exists regarding the effects of different PMSG dosages on reproductive and growth performance in this crossbreed. Previous studies on various sheep breeds: Ossimi ewes [9], Awassi [10], and Red Karaman [11] and environments have shown inconsistent responses to gonadotropin administration, emphasizing the necessity of breed- and region-specific optimization to achieve maximal reproductive efficiency.

Although PMSG (eCG) has been used for a long time to induce and synchronize estrus in sheep during the non-breeding season, the literature presents considerable variability in the recommended dosage, timing, and resulting reproductive performance. Most studies have focused on single breeds, such as Awassi, Merino, or Rahmani, under specific environmental and nutritional conditions, yielding inconsistent conclusions regarding conception rates, fecundity, and lamb survival. Limited attention has been given to crossbred genotypes, especially the Dorper × Assaf ewe, which exhibits unique physiological and adaptive traits combining high fecundity, milk potential, and environmental resilience. Furthermore, no standardized hormonal protocol currently exists for optimizing reproductive efficiency and lamb growth in Dorper × Assaf sheep reared under semi-extensive systems in Palestine’s semi-arid climate. The absence of breed-specific data on how varying PMSG doses influence conception rate, abortion incidence, and lamb performance hampers the establishment of cost-effective, welfare-oriented reproductive strategies. Therefore, identifying the optimal PMSG dosage that balances hormonal stimulation, reproductive success, and animal well-being remains a critical knowledge gap.

This study aimed to evaluate the effect of three PMSG doses (400, 500, and 600 international units [IU]) on reproductive and growth performance in Dorper × Assaf ewes during the non-breeding season under semi-extensive management conditions in Palestine. Specifically, the research sought to:

1. Determine the influence of PMSG dosage on estrous response, conception rate, fecundity, abortion rate, and lamb survival.
2. Assess the impact of PMSG levels on lamb birth and weaning weights, accounting for maternal factors such as parity and litter size.
3. Identify the optimal PMSG dose that maximizes reproductive efficiency and lamb growth while minimizing hormonal overstimulation and ensuring animal welfare. By generating breed- and environment-specific data, this study provides a scientifically validated, welfare-conscious hormonal management protocol to support sustainable small-ruminant production in semi-arid regions.

## MATERIALS AND METHODS

### Ethical approval

All experimental procedures were carried out in accordance with the Guidelines for Ethical Conduct in the Care and Use of Non-human Animals in Research [12]. As Palestine currently lacks a national Ethics Committee for animal reproduction studies, the research strictly adhered to internationally recognized standards for animal welfare and research ethics.

The experimental protocol was reviewed and supervised by the Animal and Food Genetic Resources Research Laboratory, National Agronomic Institute of Tunisia, University of Carthage, to ensure full compliance with institutional ethical standards for animal care and use. Before the commencement of the study, informed verbal consent was obtained from the farm owner to ensure transparency and voluntary participation. Throughout all experimental procedures, every effort was made to minimize animal stress, pain, and discomfort in accordance with established principles of animal welfare.

Although a formal national ethical approval number could not be issued due to the absence of a local animal ethics authority in Palestine, the experimental procedures were verified to meet the ethical and welfare criteria of the host research institution and were fully aligned with the international standards outlined by the American Psychological Association [12].

### Study period and location

The experiment was conducted in May 2024 in Nisf Jubeil, a semi-extensive sheep-farming region located in the northern West Bank, Palestine (32.28278°N, 35.22056°E; 400–571 m above sea level). The area has a Mediterranean climate, characterized by long, dry summers and cool, wet winters, which are typical of the region’s reproductive conditions for sheep. During the experimental period, the photoperiod ranged from 13.3 to 14 h. Ewes were housed in shaded pens with access to grazing pastures and were provided with concentrate feed according to standard management practices.

### Animals and management

A total of 143 healthy, non-lactating Dorper × Assaf ewes (aged 24–36 months; average body weight 55–60 kg) were selected and maintained under semi-extensive management. Only ewes with a body condition score between 3 and 4 (on a five-point scale) were included to ensure optimal reproductive performance. Random allocation to treatment groups was achieved using a computer-generated randomization table to minimize selection bias. Estrus detection personnel were blinded to treatment allocation to further reduce observer bias.

All animals were fed a standardized diet consisting of 1.5 kg of commercial concentrate (approximately 18% crude protein) and 0.5 kg of wheat hay per ewe per day, with *ad libitum* access to water.

### Estrus synchronization and hormonal treatment

Estrus synchronization and hormonal treatment protocols are summarized in Table 1. The experimental design aimed to evaluate the reproductive and growth performance of Dorper × Assaf ewes treated with different PMSG dosages.

**Table 1:** Experimental design of estrus synchronization and hormonal treatment protocol in Dorper × Assaf ewes.

Step	Description
Device and hormone	Intravaginal sponge (Sncropart sponges, CEVA Santé Animale, France) containing 30 mg of fluorogestone acetate
Duration	14 days
Exclusion	8 ewes removed due to sponge loss
Treatment groups (n = 45/group)	Intramuscular PMSG: 400, 500, and 600 IU

PMSG = Pregnant mare serum gonadotropin.

### Estrus detection and mating

Following sponge removal, estrus detection began 24 h after PMSG administration using three teaser rams. Observations were conducted every 6 h (morning and evening) over a 72-h period by two trained independent observers. Estrus was confirmed by behavioral indicators, including tail wagging, increased vocalization, frequent urination, and standing acceptance of mounting. At 45 h post-PMSG injection, fertile rams were introduced for 1 week, rotated every 24 h among treatment groups to ensure equal mating opportunities [13].

### Pregnancy diagnosis and reproductive traits

Pregnancy diagnosis was performed 28 days post-mating using a trans-abdominal ultrasonography device (Laptop Veterinary Color Doppler YR06139, Kalstein, France; 3.5–5 MHz probe). Reproductive parameters assessed included conception rate, abortion rate, lambing percentage, and fecundity.

Lambing dates, litter size, lamb sex, and birth and weaning weights were recorded for each ewe. Based on these data, the following reproductive and growth parameters were subsequently calculated using standard formulas.


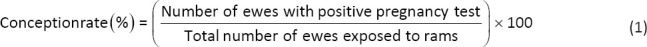



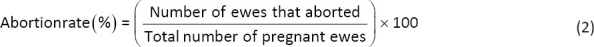



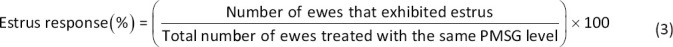



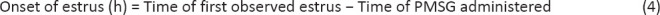



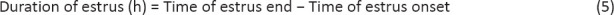



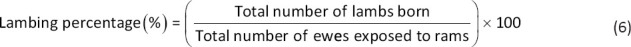



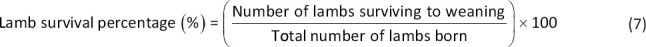



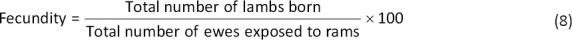



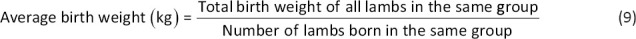



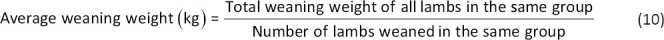


### Reproductive and growth trait calculations

The conception rate (%) was defined as the proportion of ewes confirmed pregnant among all ram-exposed ewes, representing overall mating success. The abortion rate (%) was calculated as the percentage of pregnant ewes that experienced abortion before parturition. The estrus response (%) referred to the proportion of treated ewes exhibiting behavioral signs of estrus following PMSG administration. Estrus onset (h) denoted the interval between PMSG injection and the first observed estrus, while estrus duration (h) indicated the time between estrus onset and its cessation.

The lambing percentage (%) was calculated as the total number of lambs born per ram-exposed ewe, whereas fecundity (%) represented the average number of lambs born per ewe, reflecting overall reproductive efficiency. Lamb survival (%) was expressed as the proportion of liveborn lambs that survived to weaning age. Average birth weight (kg) and average weaning weight (kg) were defined as the mean body weights of lambs at birth and at weaning, respectively, within each treatment group.

### Growth traits and lamb monitoring

Lamb birth weights were measured immediately after parturition using a calibrated digital scale (Sigma-Aldrich, USA, ±0.01 kg accuracy). Postnatal management included ensuring adequate colostrum intake, provision of creep feed, and implementation of routine vaccination schedules. Weaning was performed at 60 days of age, and lambs surviving to this stage were classified as weaning survivors. Weaning weights were recorded using the same digital scale to maintain measurement consistency across all treatment groups.

### Statistical analysis

Data were assessed for normality using the Shapiro–Wilk test and for homogeneity of variance using Levene’s test. Descriptive statistics were generated to summarize reproductive, survival, and growth traits.

A one-way analysis of variance (ANOVA) was employed to evaluate the effect of PMSG dose on reproductive performance and lamb survival, followed by Tukey’s *post hoc* test for pairwise comparisons. Results were expressed as mean ± standard error of the mean, and differences were considered statistically significant at p < 0.05. Means sharing the same letter within the same row were not significantly different (p > 0.05).

For growth traits (birth and weaning weights), a general linear model (GLM) univariate procedure was applied to evaluate the effects of PMSG dose, ewe parity, litter size, and lamb sex, according to the model:

Y_ijklm_ = μ + PMSG_i_ + Parity_j_ + Litter_k_ + Sex_l_ + e_ijklm_

- Y_ijklm_ is the observed trait value,
- μ is the overall mean,
- PMSG_i_ is the fixed effect of the PMSG dose,
- Parity_j_ is the effect of ewe parity,
- Litter_k_ general linear model is the effect of litter size,
- Sex_l_is the effect of lamb sex, and
- e_ijklm_ is the random error.

Least-square means were compared using Tukey’s test, with significance declared at p < 0.05. All statistical analyses were performed using IBM the Statistical Package for the Social Sciences Statistics version 25.0 (IBM Corp., Armonk, NY, USA).

## RESULTS

### Reproductive parameters

The effects of different PMSG doses on the reproductive performance of Dorper × Assaf ewes are summarized in Table 2. Conception rates remained consistently high across all treatment groups, with no significant differences among doses. However, the abortion rate increased markedly with rising PMSG levels, being lowest at 400 IU and highest at 600 IU. Ewes treated with 400 and 500 IU exhibited significantly lower abortion rates and superior reproductive outcomes compared with the 600 IU group.

**Table 2:** Effect of different PMSG levels on reproductive parameters and lamb survival.

PMSG level	400 IU (n = 45)	500 IU (n = 45)	600 IU (n = 45)	SEM
Conception rate	91.11	88.88	88.88	±0.31
Abortion rate	2.44^a^	5^b^	7.5^c^	±0.1
Estrus response (%)	93.67^a^	93.50^a^	90.88^b^	±0.75
Onset of estrus (h)	40.00^b^	44.00^a^	45.00^a^	±1.5
Estrus duration (h)	30.00^a^	28.00^b^	28.00^b^	±1
Lambing %	144.44	140	144.22	±0.72
Fecundity	1.62^b^	1.61^b^	1.73^a^	±0.12
Percentage of lamb survival	96.92^a^	95.08^a^	92.18^b^	±0.61

PMSG = Pregnant mare serum gonadotropin, IU = International unit, n = Sample size, SEM = Standard error of the mean, Values are reported as mean ± SEM, Values are reported as mean ± SEM. Means in the same row with similar letters (a, b, and c) are not significantly different (p > 0.05).

Estrus response was high in all groups, but ewes receiving 400 IU PMSG showed an earlier onset of estrus and a longer estrus duration, indicating enhanced reproductive efficiency at this dosage. Although lambing percentage did not differ significantly among treatments, fecundity was slightly higher in the 600 IU group, suggesting that elevated PMSG levels may increase ovulation rate but not necessarily improve overall reproductive success. The higher fecundity at 600 IU was offset by an increased abortion rate and reduced lamb survival. Lamb survival rate decreased significantly with increasing PMSG dosage, with the 400 IU group achieving the highest survival percentage.

### Lamb birth weight

The influence of PMSG dose, ewe parity, litter size, and lamb sex on birth weight is presented in Table 3. Lamb birth weight increased significantly with higher PMSG doses, being lowest in the 400 IU group and highest in the 600 IU group. Parity exerted a notable effect, with first-parity ewes producing lighter lambs compared to second- and third-parity ewes, which had similar, higher weights.

**Table 3:** Mean and SE (n = 45) of lamb birth weight according to PMSG level, parity, litter size, and lamb sex.

PMSG level			

PMSG 400 IU (n = 45)	PMSG 500 IU (n = 45)	PMSG 600 IU (n = 45)	p-value
3.42^a^± 0.18	3.55^a^ ± 0.19	3.83^b^ ± 0.18	0.01

**Parity**			

**1**	**2**	**3**	**p-value**

3.16^a^ ± 0.18	3.81^b^ ± 0.20	3.84^b^ ± 0.18	0.00

**Liter size**			

**1**	**2**	**3**	**p-value**

4.68^a^ ± 0.16	3.57^b^ ± 0.18	2.56^c^ ± 0.23	0.00

**Lamb sex**			

**Male**	**Female**		**p-value**

4.01^a^ ± 0.08	3.77^b^ ± 0.09		0.02

PMSG = Pregnant mare serum gonadotropin, IU = International unit, n = Sample size, SEM = Standard error of the mean, values are reported as mean ± SEM, Values are reported as mean ± SEM, Means in the same row with similar letters (a, b, and c) are not significantly different (p > 0.05).

Litter size showed an inverse relationship with birth weight, where singletons were heaviest, followed by twins, and triplets recorded the lowest weights. Male lambs were significantly heavier than females, reflecting the expected sex-linked growth dimorphism. These findings suggest that while increased PMSG levels and advanced parity enhance birth weight, larger litter size and female sex are associated with lower neonatal body weight, emphasizing the multifactorial nature of fetal growth regulation.

### Lamb weaning weight

The effects of PMSG dose, parity, litter size, and lamb sex on weaning weight are summarized in Table 4. Weaning weight tended to increase slightly with higher PMSG levels, being lowest in the 400 IU group and marginally higher in the 500 and 600 IU groups, though the differences were not statistically significant. Parity had no measurable effect on weaning weight, as lambs from first-, second-, and third-parity ewes exhibited similar weights.

**Table 4:** Mean and standard error (n = 45) of lamb weaning weight according to PMSG level, parity, liter size, and lamb sex.

PMSG level			

PMSG 400 IU (n = 45)	PMSG 500 IU (n = 45)	PMSG 600 IU (n = 45)	p-value
21.11^a^ ± 0.71	22.18^b^ ± 0.71	21.92^b^ ± 0.69	0.09

**Parity**			

**1**	**2**	**3**	**p-value**

21.73^a^ ± 0.69	21.39^a^ ± 0.76	22.08^a^ ± 0.70	0.36

**Liter size**			

**1**	**2**	**3**	**p-value**

24.09^a^ ± 0.62	21.88^b^ ± 0.69	19.25^c^ ± 0.86	0.00

**Lamb sex**			

**Male**	**Female**		**p-value**

23.73^a^ ± 0.32	22.02^b^ ± 0.35		0.00

PMSG = Pregnant mare serum gonadotropin, IU = International unit, n = Sample size, SEM = Standard error of the mean. Values are reported as mean ± SEM, Values are reported as mean ± SEM Means in the same row with similar letters (a, b, and c) are not significantly different (p > 0.05).

In contrast, litter size and lamb sex had pronounced effects on postnatal growth. Singletons achieved the highest weaning weights, followed by twins, while triplets showed the lowest weights, reflecting the impact of nutritional competition among littermates. Male lambs were significantly heavier than females at weaning. Overall, these results indicate that while PMSG dosage and ewe parity exert limited influence on weaning performance, litter size and sex are the principal determinants of lamb growth up to weaning.

## DISCUSSION

### Effect of PMSG dose on reproductive performance

PMSG enhances ovulation and fecundity in sheep [9], but its efficacy is highly breed-dependent, requiring breed-specific, optimized dosing strategies [10]. In this study, increasing the PMSG dose from 400 to 600 IU did not yield significant improvements in conception or lambing rates, suggesting a dose plateau beyond 400 IU. This observation aligns with earlier findings that doses above 400 IU rarely enhance fertilization or lambing outcomes [13].

The higher abortion rate recorded at 600 IU likely resulted from ovarian overstimulation and hormonal imbalance. Excessive gonadotropin stimulation can induce multiple large follicles with reduced oocyte quality, disrupt luteal function, and compromise progesterone secretion, conditions detrimental to embryo implantation and early gestation [4, 14, 15]. Similar trends were reported in Kivircik ewes, where higher PMSG doses reduced conception and lambing rates due to excessive follicular response [16], and in Rahmani ewes, where reproductive efficiency varied seasonally [17].

The 400 IU PMSG dose not only maintained reproductive performance comparable to higher doses but also provided practical advantages in terms of cost reduction and minimized hormonal side effects. Comparable findings in Afshari and Merino ewes showed that higher PMSG levels offered negligible fertility benefits but increased pregnancy loss [18, 19]. Moreover, *in vitro* studies have demonstrated that high concentrations of gonadotropins (PMSG and hCG) can impair oocyte maturation and developmental competence [20]. Thus, the 400 IU dose appears optimal for achieving a balanced endocrine response, resulting in high conception and lambing rates, low abortion incidence, and improved lamb survival.

Emerging evidence supports this low-dose efficiency model. For instance, studies on PMSG-loaded chitosan nanoparticles in Ossimi ewes reported improved fecundity at lower encapsulated doses (300 IU) compared with conventional higher doses [21]. Similarly, Anatolian Merino sheep showed comparable lambing rates between 300 IU and 600 IU treatments, reinforcing the value of low-hormone reproductive strategies that achieve high efficiency with minimal endocrine disruption [22–24].

### Determinants of lamb birth weight

The effects of PMSG dose, ewe parity, litter size, and sex on lamb birth weight observed in Dorper × Assaf ewes align with established physiological patterns in small ruminants. Lamb birth weight is influenced by complex interactions among genetic, maternal, and environmental factors, underscoring the need to integrate these factors into breeding and management strategies [25].

The observed increase in birth weight with higher PMSG doses corroborates findings in Akkaraman crossbred ewes, where 700 IU PMSG significantly increased estrus and twinning rates [26]. However, the benefits of such high doses may be offset by metabolic strain and reproductive complications. Parity significantly affected birth weight, as first-parity ewes produced lighter lambs compared to multiparous ewes, consistent with observations in Awassi and Assaf breeds [27]. Maternal maturity, improved uterine capacity, and better nutrient partitioning likely explain these differences.

Litter size was inversely correlated with birth weight (p < 0.01), with singletons being heavier than twins and triplets. This pattern aligns with the findings of Azzam *et al*. [28] and Van Donkersgoed [29], who associated increased litter size with lower neonatal weight and higher mortality risk. This trade-off underscores the biological balance between prolificacy and neonatal viability, a central consideration in sheep breeding. Furthermore, male lambs were consistently heavier than females, confirming the well-documented sex-linked growth dimorphism observed across multiple breeds, including the Palestinian Assaf [27]. Collectively, these results highlight the multifactorial determinants of lamb birth weight and their implications for breeding strategies aimed at optimizing productivity and survivability.

### Factors influencing lamb weaning weight

Lamb weaning weight was primarily determined by litter size and sex, while PMSG dose and parity exerted minimal influence. The slight, non-significant increase in weaning weight with higher PMSG levels (400–600 IU) suggests that moderate follicular stimulation may enhance early growth but has diminishing effects by weaning age. These findings are consistent with those of Gardner *et al*. [30], who emphasized that maternal environment and litter size have a stronger effect on postnatal growth than parity.

Singleton lambs demonstrated higher weaning weights than twins and triplets, reflecting the nutritional competition effect among littermates both *in utero* and postnatally. The reduced weights observed in multiple births likely result from limited maternal resources and increased sibling competition for milk [30].

Sex differences were also evident, with male lambs significantly outperforming females in preweaning growth. This dimorphism is attributed to hormonal and metabolic factors that enhance muscle accretion and energy utilization in males [26, 27]. Similar patterns reported in Palestinian Assaf and Awassi populations confirm that sex-linked growth variation is an intrinsic physiological characteristic rather than a management artifact.

Overall, the results indicate that PMSG dosage beyond 400 IU offers no additional benefit for postnatal growth, while litter size and sex remain the dominant determinants of lamb performance. Integrating moderate hormonal protocols with genetic selection, nutritional optimization, and welfare-conscious management can enhance both productivity and sustainability in small ruminant systems.

## CONCLUSION

This study demonstrated that administering 400 IU of PMSG to Dorper × Assaf ewes during the non-breeding season provides the most effective balance between reproductive efficiency, lamb survival, and animal welfare under semi-extensive conditions in Palestine. Ewes treated with 400 IU exhibited high conception and lambing rates, a low abortion incidence (2.44%), and the highest lamb survival (96.9%), while maintaining satisfactory birth (3.42 kg) and weaning weights (21.11 kg). Increasing the dose to 500 or 600 IU did not improve reproductive or growth outcomes and was associated with a higher abortion rate and a slight reduction in lamb survival. Growth performance was mainly influenced by litter size and lamb sex, with singletons and males showing superior birth and weaning weights compared to multiples and females.

The practical implication of these findings is that moderate hormonal stimulation with 400 IU PMSG effectively enhances fertility and fecundity while minimizing health risks and costs. This dosage avoids the detrimental effects of ovarian overstimulation, reduces veterinary interventions, and aligns with animal welfare principles. Hence, the proposed protocol serves as a cost-effective and welfare-conscious reproductive management strategy, particularly suitable for sheep-rearing systems in semi-arid regions.

A major strength of this study lies in its controlled experimental design, which included random allocation, blinded estrus detection, and robust statistical validation through ANOVA and GLM models. It represents the first systematic evaluation of PMSG dosage optimization for Dorper × Assaf ewes, combining reproductive and growth performance indicators to inform practical management strategies. However, the findings are limited to a single reproductive season and one agro-climatic region. In addition, hormonal or metabolic profiling was not included, which could have provided mechanistic insights into dose-dependent physiological effects.

Future research should investigate the potential of nanoparticle-based or slow-release PMSG formulations to prolong hormonal efficacy while reducing dosage and assess lower-dose or hormone-free synchronization protocols integrated with nutritional or management interventions. Long-term, multi-seasonal studies across different climatic zones and crossbred genotypes would further refine and validate these findings.

The 400 IU PMSG protocol offers an optimal, sustainable, and welfare-oriented approach for improving reproductive efficiency and lamb survival in Dorper × Assaf ewes. These outcomes reinforce the concept that “less is more” in reproductive hormone management – achieving productivity without compromising health or ethics.

## DATA AVAILABILITY

All data generated during the study are included in the manuscript.

## AUTHORS’ CONTRIBUTIONS

WH, MS, SK, and IB: Conceptualized and designed the methodology, collected data, performed statistical analysis, conducted the literature review, visualizations, and drafted and edited the manuscript. IB, MS, and MK: Analyzed data, interpreted results, and critically revised the manuscript. NM: Supervised the study and administrated the project. All authors have read and approved the final manuscript.

## References

[1] Duman M, Şekeroğlu A, Aksoy Y (2024). Investigation of some fertility and growth traits of Akkaraman sheep under breeder condition in Altunhisar District of Niğde Province. Turk. J. Agric. Food Sci. Technol.

[2] Husein M.Q, Kridli R.T (2002). Effects of progesterone sponges in Awassi ewes. Asian Australas. J. Anim. Sci.

[3] Halaweh W, Khnissi S, Ben Souf I, Salman M, M'Hamdi N (2025). Impact of mating methods and semen preservation on reproductive and growth performances in Palestinian Assaf sheep. Biology (Basel).

[4] Abecia J.A, Forcada F, González-Bulnes A (2012). Hormonal control of reproduction in small ruminants. Anim. Reprod. Sci.

[5] Hameed N, Khan M.I.U.R, Zubair M, Andrabi S.M.H (2021). Approaches of estrous synchronization in sheep:Developments during the last two decades:A review. Trop. Anim. Health Prod.

[6] Arya D, Goswami R, Sharma M (2023). Estrous synchronization in cattle, sheep and goat. Multidiscip. Rev.

[7] Ahmed M, Abdallah J.M (2012). Comparison of milk yield and reproductive performance of sheep breeds in the West Bank, Palestine. Najah Univ. J. Res. Nat. Sci.

[8] Ojango J.M.K, Okpeku M, Osei-Amponsah R, Kugonza D.R, Mwai O, Changunda M.G.G, Rege J.E.O (2023). Dorper sheep in Africa:A review of their use and performance in different environments. CABI Rev.

[9] Ali A (2007). Effect of time of eCG administration on follicular response and reproductive performance of FGA-treated Ossimi ewes. Small Rumin. Res.

[10] Emsen E, Yaprak M (2006). Effect of controlled breeding on the fertility of Awassi and Red Karaman ewes and the performance of the offspring. Small Rumin. Res.

[11] Zonturlu A.K, Özyurtlu N, Kaçar C (2009). Effect of different doses PMSG on estrus synchronization and fertility in Awassi Ewes synchronized with Progesterone during the transition period [Geçişdöneminde progesteron ile senkronize edilen İvesi koyunlarda PMSG'nin farklıdozlarının östrus senkronizasyonu ve fertilite üzerine etkisi]. Kafkas Univ. Vet. Fak. Derg.

[12] American Psychological Association (2021). Guidelines for Ethical Conduct in the Care and Use of Nonhuman Animals in Research.

[13] Fallah-Rad A.H, Farzaneh N (2007). Effect of CIDR and different doses of PMSG on pregnancy and lambing rate out of breeding season in Balouchi ewes. J. Anim. Vet. Adv.

[14] Gonzalez-Bulnes A, Veiga-Lopez A, Garcia P, Garcia-Garcia R.M, Ariznavarreta C, Sanchez M.A, Tresguerres J.A, Cocero M.J, Flores J.M (2005). Effects of progestagens and prostaglandin analogues on ovarian function and embryo viability in sheep. Theriogenology.

[15] Spencer T.E, Johnson G.A, Bazer F.W, Burghardt R.C (2004). Implantation mechanisms:Insights from the sheep. Reproduction.

[16] Doğan İ, Nur Z (2006). Different estrous induction methods during the non-breeding season in Kivircik ewes. Vet. Med.

[17] AbdEl-Rafaa L, Ashmawy T, AbdEl-Khalek A (2022). Reproductive performance of Rahmani ewes in breeding and non-breeding seasons according to different hormonal protocols. Egypt. J. Sheep Goats Sci.

[18] Karimi B, Ebtekar M (2018). Effects of pregnant mare serum gonadotropin (PMSG) administration on lambing rate of Afshari ewes. Int. J. Anim. Sci.

[19] Lebata P.S, Dawuda P.M, Molapo S (2024). Oestrus synchronization in Merino sheep using intravaginal sponges, Estrumate PMSG on oestrus activity. Indian J. Anim. Reprod.

[20] Baiomy F.M, Kamel A.M, Hussein A.F, Hassanin S.H (2025). The impact of maturation medium supplemented with PMSG and HCG hormones on *in vitro* maturation of ovine oocytes. J. Appl. Vet. Sci.

[21] Shehabeldin A.M, Salama M.S, Omar M.E.A, AbdEl-Rafaa L.A, Ashour M.A, Hassan M.A.E, El-Shereif A.A, Abdelmegeid M, Shukry M, Elolimy A.A (2025). Improving the breeding capabilities of short-term estrus synchronized Ossimi sheep using pregnant mare serum gonadotropin loaded chitosan-nanoparticles. Front. Vet. Sci.

[22] Sirin E (2022). The effect on fertility of using different doses of PMSG in Anatolian Merino sheep. Black Sea J. Agric.

[23] Laclef E, González-García E, Debus N, Taillandier P, De Boissieu C, Morin E, Tichit M (2024). Inseminate without hormonal treatment in dairy sheep farms:Exploring the consequences on the sustainability of several contrasted production systems. Animal.

[24] AL-Jaryan I.L, AL-Thuwaini T.M, Merzah L.H, Alkhammas A.H (2023). Reproductive physiology and advanced technologies in sheep reproduction. Rev. Agric. Sci.

[25] Jawasreh K, Ismail Z.B, Iya F, Castaneda-Bustos V.J, Valencia-Posadas M (2018). Genetic parameter estimation for pre-weaning growth traits in Jordan Awassi sheep. Vet. World.

[26] Akoz M, Bülbül B, Ataman M.B, Dere S (2006). Induction of multiple births in Akkaraman cross-bred sheep synchronized with short duration and different doses of progesterone treatment combined with PMSG outside the breeding season. Bull. Vet. Inst. Pulawy.

[27] Salman M, Ben Souf I, Khnissi S, Halaweh W, M'Hamdi N (2024). Estimate of genetic parameters for pre-weaning growth traits and Kleiber ratio in Palestinian sheep breeds. Agriculture.

[28] Azzam K.A, Tabbaa M.J, Al-Atiyat R.M, Titi H.H (2025). Phenotypic characteristics and factors associated with Assaf lamb body weight and morphology. Qeios.

[29] Van Donkersgoed J (2025). Ewe litter size and lamb birth weight:Effects on lamb health, performance, and carcass traits. Can. Vet. J.

[30] Gardner D.S, Buttery P.J, Daniel Z, Symonds M.E (2007). Factors affecting birth weight in sheep:Maternal environment. Reproduction.

